# Phenotypic diversity and drug susceptibility of *Trypanosoma cruzi* TcV clinical isolates

**DOI:** 10.1371/journal.pone.0203462

**Published:** 2018-09-05

**Authors:** Luz P. Quebrada Palacio, Mariela N. González, Yolanda Hernandez-Vasquez, Alina E. Perrone, Adriana Parodi-Talice, Jacqueline Bua, Miriam Postan

**Affiliations:** 1 Consejo Nacional de Investigaciones Científicas y Técnicas (CONICET), Buenos Aires, Argentina; 2 Departamento de Investigación, Instituto Nacional de Parasitología “Dr. Mario Fatala Chabén”, Buenos Aires, Argentina; 3 Unidad de Biología Molecular, Institut Pasteur de Montevideo, Sección Genética, Facultad de Ciencias, Universidad de la República, Montevideo, Uruguay; Instituto Butantan, BRAZIL

## Abstract

*Trypanosoma cruzi* is a genetically heterogeneous group of organisms that cause Chagas disease. It has been long suspected that the clinical outcome of the disease and response to therapeutic agents are, at least in part, related to the genetic characteristics of the parasite. Herein, we sought to validate the significance of the genotype of *T*. *cruzi* isolates recovered from patients with different clinical forms of Chagas disease living in Argentina on their biological behaviour and susceptibility to drugs. Genotype identification of the newly established isolates confirmed the reported predominance of TcV, with a minor frequency of TcI. Epimastigote sensitivity assays demonstrated marked dissimilar responses to benznidazole, nifurtimox, pentamidine and dihydroartemisinin *in vitro*. Two TcV isolates exhibiting divergent response to benznidazole in epimastigote assays were further tested for the expression of anti-oxidant proteins. Benznidazole-resistant BOL-FC10A epimastigotes had decreased expression of Old Yellow Enzyme and cytosolic superoxide dismutase, and overexpression of mitochondrial superoxide dismutase and tryparedoxin- 1, compared to benznidazole-susceptible AR-SE23C parasites. Drug sensitivity assays on intracellular amastigotes and trypomastigotes reproduced the higher susceptibility of AR-SE23C over BOL-FC10A parasites to benznidazole observed in epimastigotes assays. However, the susceptibility/resistance profile of amastigotes and trypomastigotes to nifurtimox, pentamidine and dihydroartemisinin varied markedly with respect to that of epimastigotes. C3H/He mice infected with AR-SE23C trypomastigotes had higher levels of parasitemia and mortality rate during the acute phase of infection compared to mice infected with BOL-FC10A trypomastigotes. Treatment of infected mice with benznidazole or nifurtimox was efficient to reduce patent parasitemia induced by either isolate. Nevertheless, qPCR performed at 70 dpi revealed parasite DNA in the blood of mice infected with AR-SE23C but not in BOL-FC10A infected mice. These results demonstrate high level of intra-type diversity which may represent an important obstacle for the testing of chemotherapeutic agents.

## Introduction

It is estimated that over 7–8 million people are currently infected with *Trypanosoma cruzi*, the causative agent of Chagas disease [1]. Infection by this parasite results in a broad spectrum of clinical outcomes, ranging from a mild, self-resolving acute illness with low mortality rate to a potentially life-threatening cardiomyopathy, digestive and/or neurological abnormalities in up to 30% of chronically infected individuals. Chagas disease causes approximately 12,000 deaths per year [2]. Current therapy against *T*. *cruzi* relies on the nitroimidazole benznidazole (BZ) and the nitrofuran nifurtimox (NX), which are efficient to control parasitism during the acute phase of infection; however, studies on their effectiveness in the chronic phase have yielded inconsistent results [3,4].

*T*. *cruzi* is a genetically heterogeneous species of protozoan parasites transmitted by triatomine bugs distributed throughout the Americas. The large variety of host and vector species involved in the natural transmission of trypanosomes and their circulation in different eco-epidemiological settings have been held responsible for the generation of structurally different populations of parasites [5]. Following the introduction of a consensus nomenclature system for *T*. *cruzi*, namely discrete typing units (DTUs I-VI) [6,7], numerous studies have aimed to characterize the geographical distribution of *T*. *cruzi* lineages in humans and other mammals as well as their association with clinical manifestations of Chagas disease [8–11]. A substantial amount of experimental data relating the genotype of the parasite with biological and biochemical parameters such as parasitemia levels, tissue tropism, immune response and susceptibility to drugs have also become available, mostly for stocks collected decades ago from different sources and countries [12–17]. In this study, we sought to assess the genotype of clinically relevant *T*. *cruzi* isolates obtained from infected humans of Argentina and evaluate their biological characteristics and susceptibility to conventional and investigational drugs. The results of parasite DTU identification analysis reproduced the predominant circulation of TcV reported by studies genotyping whole blood, with a minor frequency of TcI [11]. By testing drug susceptibility *in vitro*, we succeeded in documenting high intra-type diversity among TcV isolates, with some of them being resistant to the compounds tested. Our observations of large disparities in drug susceptibility responses are not supported by the current DTU phylogenetics classification and highlight the importance of investigating intra-type diversity in eco-epidemiological, clinical and pharmacological studies of *T*. *cruzi*.

## Results

**Fig 1:**
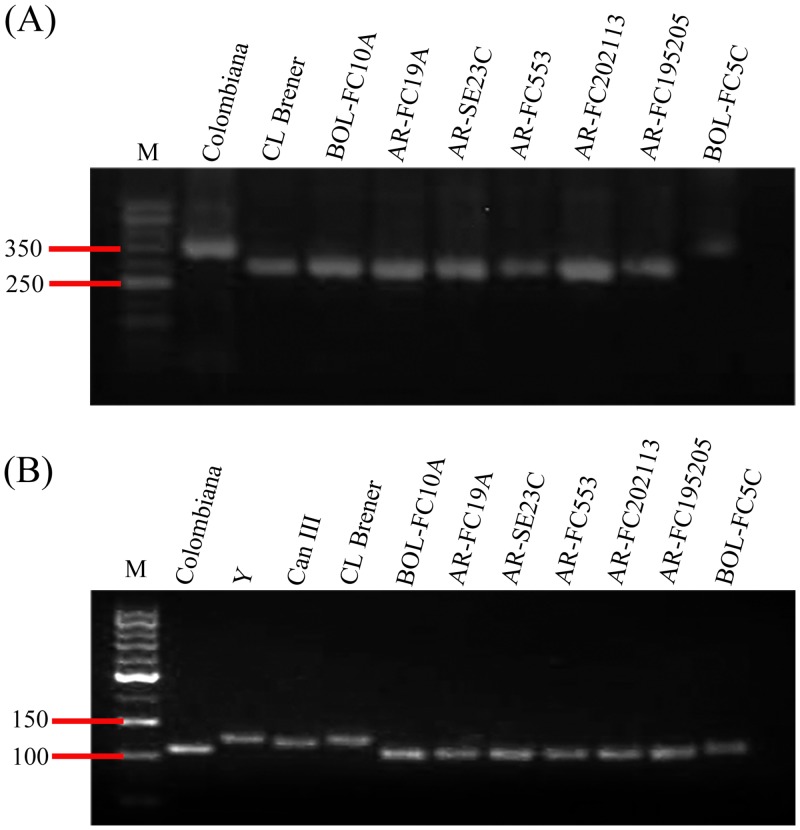
Genotyping of *Trypanosoma cruzi* isolates obtained from Chagas disease patients. **(A)** PCR amplification products of the non-transcribed spacer of the mini-exon genes with primers TC, TC1 and TC2 that identify TcI (350 bp), TcII, TcV and TcVI (300 bp) and TcIII and TcIV (no amplification). **(B)** PCR amplification products of the D7 divergent domain of S24α rRNA with primers D71 and D72 that identify TcI, TcIII and TcV (110 bp), TcIV (120, 125 or 130 bp), TcII and TcVI (125bp). *Trypanosoma cruzi* strains Colombiana, CL Brener, Y, and CanIII were included as genotyping controls.

In this study, we first established a collection of *T*. *cruzi* isolates with parasites recovered from the peripheral blood of 5 chronically infected adult Chagas disease patients and 2 acutely infected children living in Argentina (2 male and 5 female). The geographic origin, age and clinical status of the subjects from whom the parasite isolates were derived are summarized in Table 1. Amplification of the non-transcribed spacer of the mini-exon genes in epimastigotes generated products of 350 bp (TcI) in 1/7 isolates (14.28%) and 300 bp (TcII, TcV and TcVI) in 6/7 (85.71%) [18,19]. Amplification of 24Sα rRNA was used to resolve the genotype of the six isolates with 300 bp amplicons in the first PCR as TcV (Table 1 and Fig 1). The occurrence of TcV isolates originated in acute and chronic infections of different severities challenges the notion that clinical outcome of Chagas disease is related to lineage-specific parasites [20].

**Table 1:** Characteristics of the Chagas disease patients with *Trypanosoma cruzi* positive hemoculture and parasite genotype identification.

Isolate	Geographic origin^a^	Age^b^	Clinical status	DTU
BOL-FC5C	Potosi, Bolivia	42 y	Abnormal ECG	TcI
BOL-FC10A	Villazón, Bolivia	31 y	Asymptomatic	TcV
AR-FC19A	Buenos Aires, Argentina	51 y	Asymptomatic	TcV
AR-SE23C	Santiago del Estero, Argentina	57 y	Chagas cardiomyopathy	TcV
AR-FC553	Entre Ríos, Argentina	64 y	Asymptomatic	TcV
AR-FC202113	Buenos Aires, Argentina^c^	19 mo	Acute, congenital	TcV
AR-FC195205	Buenos Aires, Argentina^c^	7 mo	Acute, congenital	TcV

^a^Locality and Country of birth.

^b^Expressed in years (y) and months (mo).

^c^Children born to *T*. *cruzi*-infected mothers from Province Chaco, Argentina

**Fig 2:**
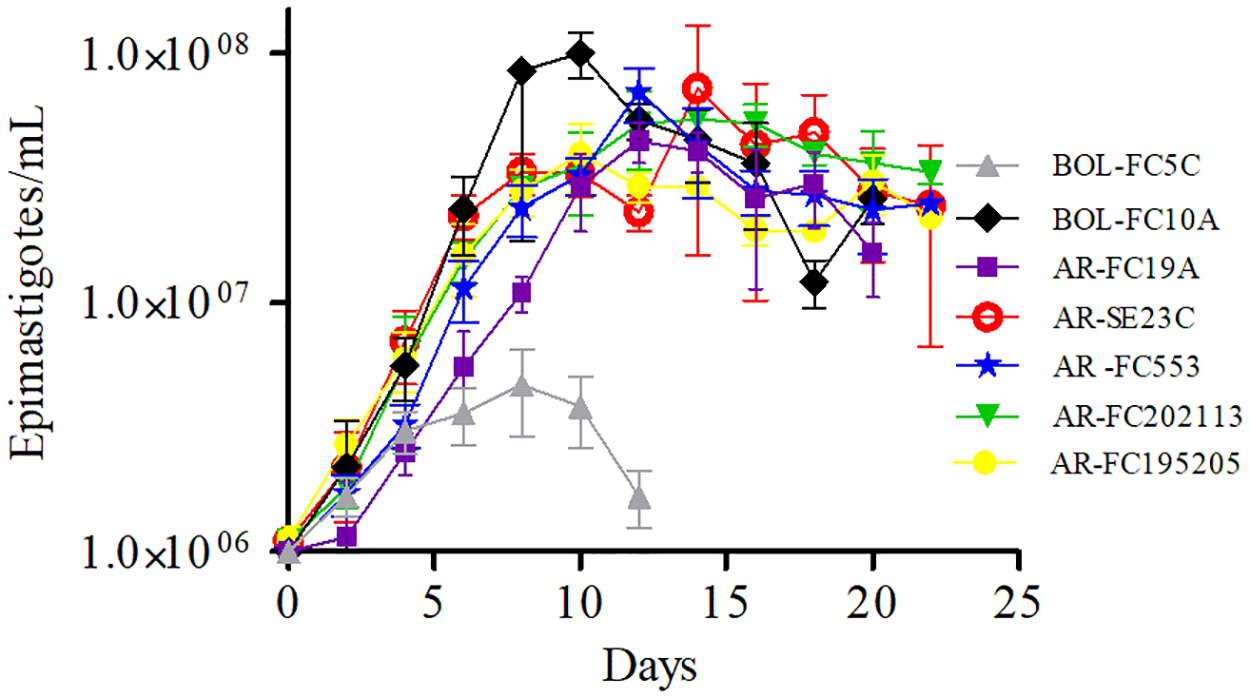
Growth curves of *Trypanosoma cruzi* isolates recovered from humans. Epimastigote cultures were set up in LIT media at initial concentration of 10^6^ epimastigotes/mL and incubated at 28°C. Parasite density was estimated daily with a Neubauer’s chamber during 3 weeks.

Then, we assessed the behavior of the clinical isolates in axenic cultures. A typical exponential curve that reached the stationary phase at 8–12 days of culture was obtained for TcV isolates; the growth curve of TcI BOL-FC5C isolate was atypical in that the exponential growth phase was followed by a rapid decline in parasite density (Fig 2). The estimated doubling time of TcI BOL-FC5C (90.85 h) was significantly higher compared to that of TcV isolates (range 35.56–52.34 h; p<0.05); no significant differences in doubling time between TcV isolates were observed. The density of parasites attained by TcI BOL-FC5C cultures at the end of the exponential phase was significantly lower than that of TcV isolates cultures (p<0.05). There were also significant differences in maximum parasite density among TcV cultures (p<0.05). No correlation between doubling time and maximum parasite density was found.

Afterward, we tested the sensitivity of epimastigotes to conventional drugs BZ and NX, and investigational compounds pentamidine (PENT) and dihidroartemisinin (DHA). The reference strain TcI Sylvio-X10/4, originated from a human infection, was included as susceptibility control to BZ [21] and for comparison with our newly established TcI isolate. Epimastigote sensitivity assays revealed remarkable dissimilar intra-DTU V and intra-DTU I susceptibility to each drug (p<0.05; Table 2 and S1 Fig). To establish the susceptibility/resistance phenotype, we applied the hit selection criteria for Chagas disease proposed by Katsuno et al (IC_50_< 10 μM) [22] and found that AR-FC195205 and BOL-FC10A epimastigotes were resistant to BZ; these isolates were also resistant to NX or PENT, respectively. None of the isolates was found to be susceptible to DHA in epimastigote sensitivity assays.

**Table 2:** Susceptibility of *Trypanosoma cruzi* epimastigotes to conventional chemotherapeutic agents and investigational compounds.

DTU	*T*.*cruzi* isolate	Benznidazole	Nifurtimox	Pentamidine	Dihydroartemisinin
TcI	Sylvio-X10/4	1.09±0.2 (4.2±1.0)	0.13±0,0 (0.47±0.1)	1.60±1.0 (2.68±1.7)	10.25±1.9 (35.96±6.8) ^a^
	BOL-FC5C	2.49±0.4 (9.56±1.8)	2.33±0.6 (8.13±2.3)	3.86±1.1 (6.49±1.8)	17.03±3.4 (59.77±12.1) ^a^
	P (*t-test*) =	0.0002	0.0001	0.0053	0.0018
TcV	BOL-FC10A	19.03±2.1 (70.6±8.1) ^a^	1.51±0.1 (5.25±0.6)	6.26±1.1 (10.5±1.8) ^a^	17.64±1.8 (61.93±6.6) ^a^
	AR-FC19A	1.24±0.2 (4.79±0.9)	0.94±0.1 (3.27±0.6)	2.13±0.3 (3.59±0.5)	13.39±1.6 (47.02±5.6) ^a^
	AR-SE23C	0.87±0.2 (3.35±1.0)	1.45±0.2 (5.06±0.8)	1.63±1.0 (2.74±1.7)	8.17±2.8 (28.70±9.9) ^a^
	AR -FC553	1.99±0.2 (7.66±0.8)	1.59±0.1 (5.54±0.3)	1.89±0.8 (3.18±1.4)	8.21±1.7 (28.85±5.9) ^a^
	AR-FC202113	0.62±0.2 (2.39±1.0)	0.34±0.1 (1.21±0.1)	2.19±1.4 (3.68±2.5)	6.87±2.1 (24.14±7.4) ^a^
	AR-FC195205	10.57±1.1 (40.62±4.3) ^a^	4.05±0.6 (14.11±2.4) ^a^	2.43±0.4 (4.09±0.7)	13.41±0.7 (47.08±2.7) ^a^
	P (ANOVA) =	0.0001	0.0001	0.0001	0.0001

Data represent mean ± SD IC_50_ values, expressed as μg/mL (μM). Epimastigote cultures were treated with anti-*T*. *cruzi* compounds (0.1–80 μg/mL) during 72 h. P values were determined by means of *t-test* for comparing TcI isolates and One-way ANOVA followed by Bonferroni for TcV isolates.

^a^Cut-off for resistance was set at IC_50_ >10μM [22].

**Fig 3:**
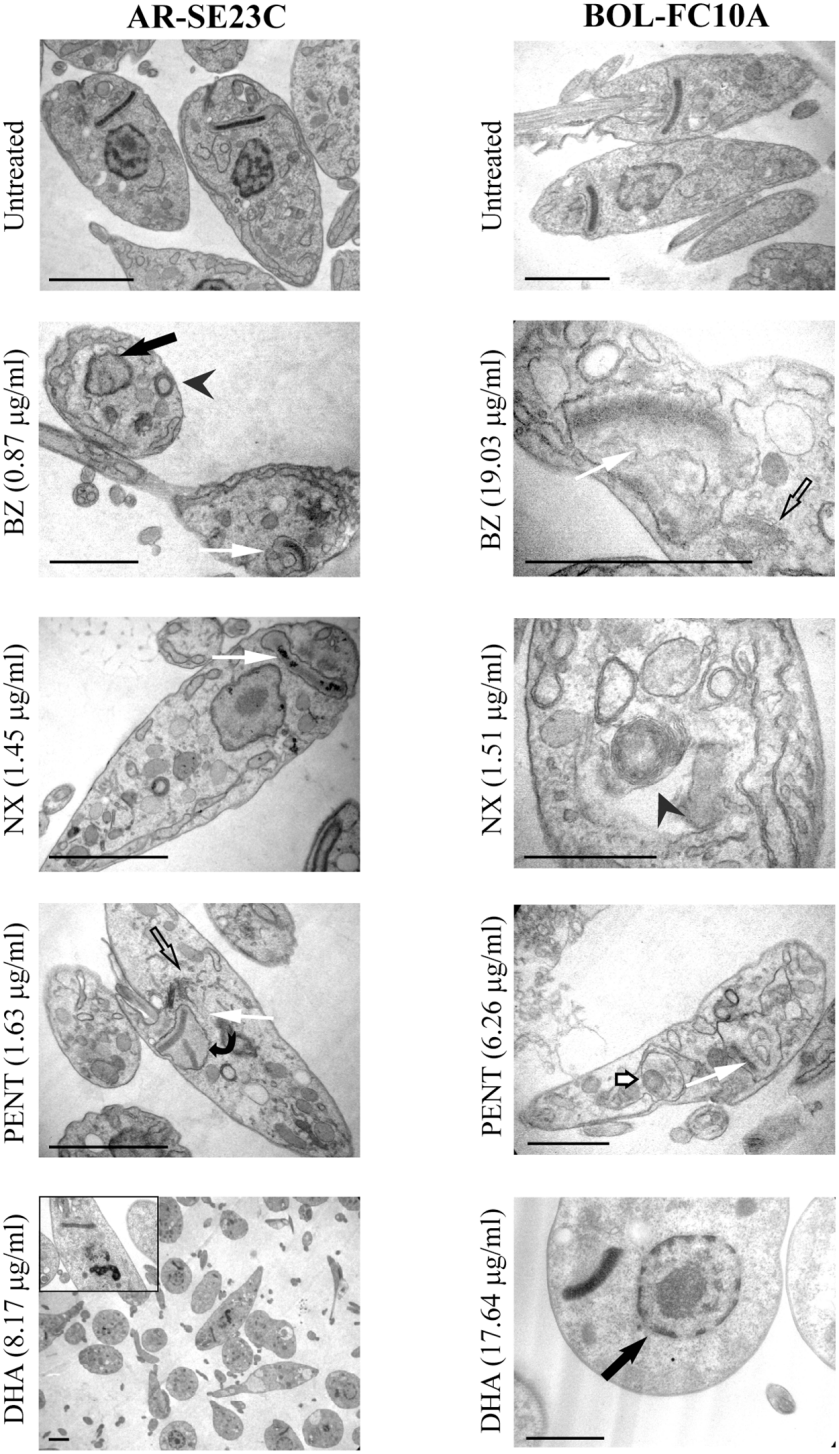
Ultrastructural changes in epimastigotes treated with conventional and investigational anti- *Trypanosoma cruzi* compounds. AR-SE23C and BOL-FC10A epimastigote cultures were incubated with IC_50_ doses for each compound and isolate during 72 h at 28°C; untreated epimastigotes were included as controls. Note the swelling of the mitochondrion with kDNA degradation (white arrow), myelin-like structures (arrowhead), disorganization of nuclear chromatin and nuclear membrane disruption (black arrow), the irregular replication of kDNA (curved arrow), disorganization of Golgi complex cisternae (unfilled black arrow) and autophagosomal structures (short white arrow). Bars represent 2.5 μm.

To identify possible morphological changes induced by the compounds on epimastigotes, two TcV isolates derived from chronically infected adult patients exhibiting divergent sensitivity to BZ in epimastigote assays (AR-SE23C susceptible and BOL-FC10A resistant) were treated with the respective IC_50_ doses of each compound during 72 h at 28°C and evaluated at TEM (Fig 3). The most frequently observed changes include swelling of the mitochondrion with membrane damage and kDNA degradation, disruption of the nuclear membrane with disorganization of the chromatin, abnormal-shape reservosomes and acidocalcisomes, vacuole formation and large vesicles in the matrix, common to both isolates and the four compounds tested. Enlargement of Golgi cisternae and rare myelin-like structures were observed in parasites treated with BZ, NX and PENT, and autophagolysosomes in BZ and PENT- treated parasites. Rare abnormal kinetoplastic DNA replication was detected in PENT-treated AR-SE23C epimastigotes; spherical epimastigotes with rare swelling of the mitochondrion were observed in parasites treated with DHA.

**Fig 4:**
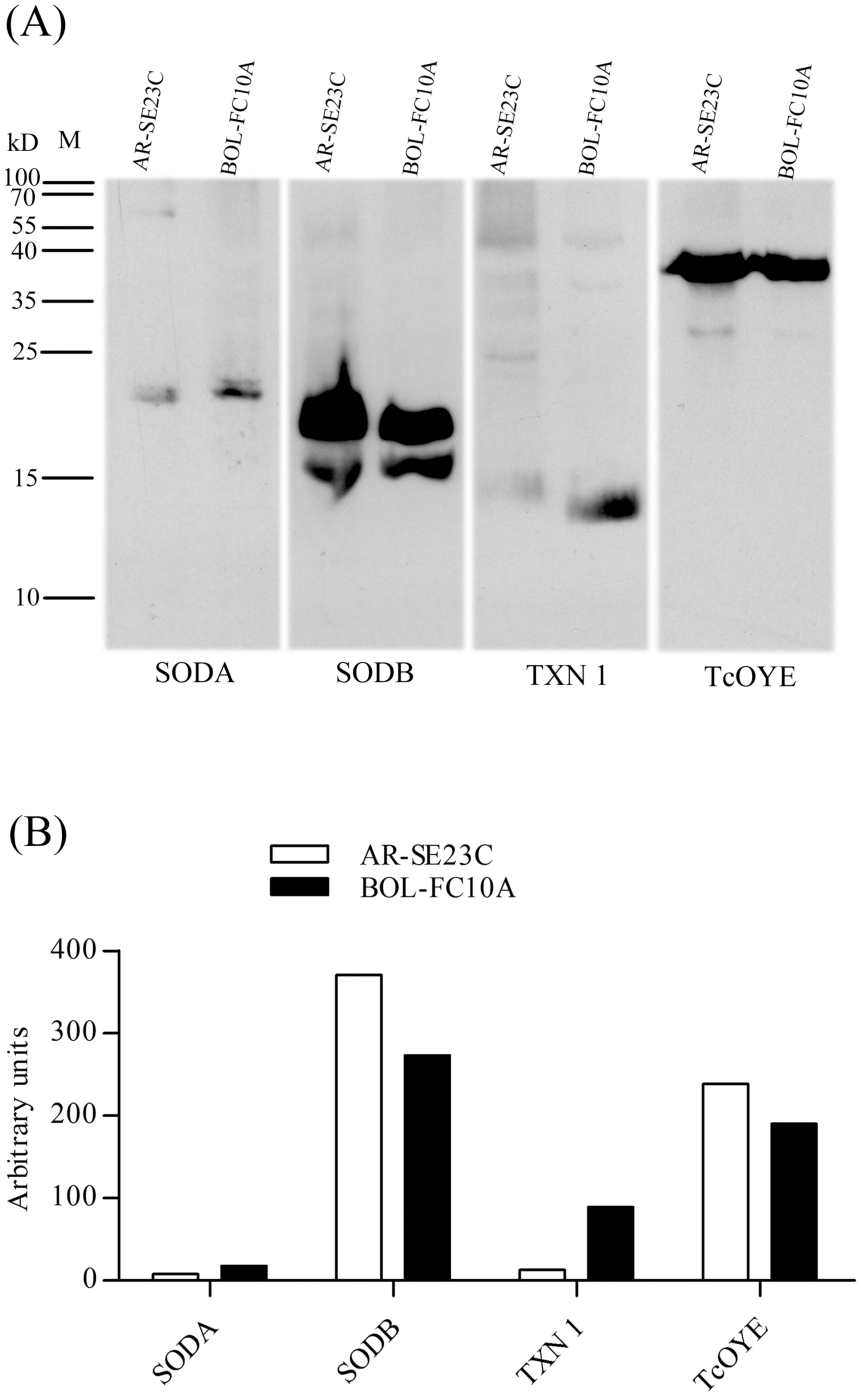
Expression of anti-oxidant proteins in *Trypanosoma cruzi*. **(A)** Western blotting for *T*. *cruzi* SODA, SODB, TXN 1 and TcOYE in BZ-susceptible AR-SE23C and BZ-resistant BOL-FC10A epimastigotes. **(B)** Densitometric analysis; bars represent the relative intensity values estimated by densitometry using Scion Imaging Software; representative assay of 2 independent experiments.

We also measured the expression of enzymes involved in oxidant detoxification of *T*. *cruzi* in BZ-susceptible AR-SE23C and BZ-resistant BOL-FC10A epimastigotes. The expression of TcOYE and SODB, reportedly associated with susceptibility to BZ [23,24], were 1.25 and 1.36 -fold increased in AR-SE23C parasites, respectively, compared to that of BOL-FC10A parasites. In contrast, the expression of SODA and TXN 1, associated with resistance to BZ [12,25] were 2.23 -fold and 6.84 -fold higher in BOL-FC10A parasites, respectively, compared to AR-SE23C parasites (Fig 4; S2 Fig). These results support a possible relationship between drug susceptibility/resistance phenotype and *T*. *cruzi* epimastigote anti-oxidant protein expression profile.

We next tested the *in vitro* susceptibility/resistance profile of AR-SE23C and BOL-FC10A intracellular forms to the compounds. Drug sensitivity assays using infected peritoneal mouse macrophages (PMØ) cultures showed that the IC_50_ of BZ for AR-SE23C amastigotes was significantly lower than that for BOL-FC10A amastigotes (p<0.05; Table 3). Conversely, the IC_50_ of NX and PENT for AR-SE23C amastigotes were significantly higher compared to that of BOL-FC10A (p<0.05); no differences between IC_50_ values of DHA for the two isolates were found. Considering the Katsuno hit criteria [22], AR-SE23C amastigotes were susceptible to BZ, NX and PENT while BOL-FC10A amastigotes were resistant to BZ; amastigote forms of both isolates were resistant to DHA.

**Table 3:** Susceptibility of *Trypanosoma cruzi* AR-SE23C and BOL-FC10A amastigote and trypomastigote forms to conventional and investigational compounds.

*T*.*cruzi* isolate		Benznidazole	Nifurtimox	Pentamidine	Dihydroartemisinin
Amastigotes	AR-SE23C	1.48±0.4 (5.68±1.5)	0.36±0.0 (1.25±0.0)	2.47±0.4 (4.14±0.6)	13.5±3.2 (47.38±11.2)^a^
	BOL-FC10A	3.07±0.7 (11.78±2.6) ^a^	0.05±0.0 (0.17±0.0)	0.68±0.2 (1.14±0.3)	11.9±1.0 (41.76±3.5) ^a^
	P (*t-test)* =	0.0476	0.0021	0.0037	0.5562
Trypomastigotes	AR-SE23C	0.40±0.1 (1.53±0.3)	0.03±0.0 (0.10±0.0)	0.43±0.14 (0.72±0.1)	0.31±0.0 (1.08±0.0)
	BOL-FC10A	18.02±1.8 (69.19±6.9) ^a^	11.56±1.6 (40.22±5.5) ^a^	10.63±2.4 (17.85±4.0) ^a^	58.42±0.9 (205.05±3.1) ^a^
	P (*t-test)* =	< 0.0001	< 0.0001	< 0.0001	< 0.0001

Data represent mean ± SD IC_50_ values, expressed as μg/mL (μM). Infected macrophage cultures and Vero cell culture- derived trypomastigotes were treated with anti-*T*. *cruzi* compounds (0.01–100 μg/mL) during 72 h. P values represent statistical significance between the compounds IC_50_ values for each isolate and stage (*t-test*).

^a^Cut-off for resistance was set at IC_50_ >10μM [22].

Cytotoxicity assays performed on uninfected PMØ cultures showed that the concentrations of BZ, NX, DHA and PENT required to reduce 50% of viable cells (CC_50_) were 4.84 mM (1.26 mg/mL), 4.84 mM (1.39 mg/mL), 3.30 mM (0.94 mg/mL) and 0.14 mM (0.087mg/mL), respectively. While the Selectivity Index (SI) of BZ, NX and DHA resulted >50 for AR-SE23C and BOL-FC10A amastigotes, the SI of PENT was >50 for BOL-FC10A but <50 for AR-SE23C amastigotes (SI = 35.6).

Drug sensitivity assays performed on trypomastigotes released from Vero cell cultures showed significantly higher IC_50_ values of BZ, NX, PENT and DHA against BOL-FC10A compared to that for AR-SE23C trypomastigotes (Table 3). While IC_50_ values against AR-SE23C trypomastigotes were below 10μM, IC_50_s against BOL-FC10A were all above that concentration. Thus, AR-SE23C trypomastigotes were considered susceptible and BOL-FC10A trypomastigotes resistant to the compounds. The four compounds had a SI >50 against AR-SE23C trypomastigotes, as well as BZ and NX against BOL-FC10A trypomastigotes; the SI of PENT and DHA against BOL-FC10A trypomastigotes was much lower (SI = 8.18 and SI = 16.09, respectively).

**Fig 5:**
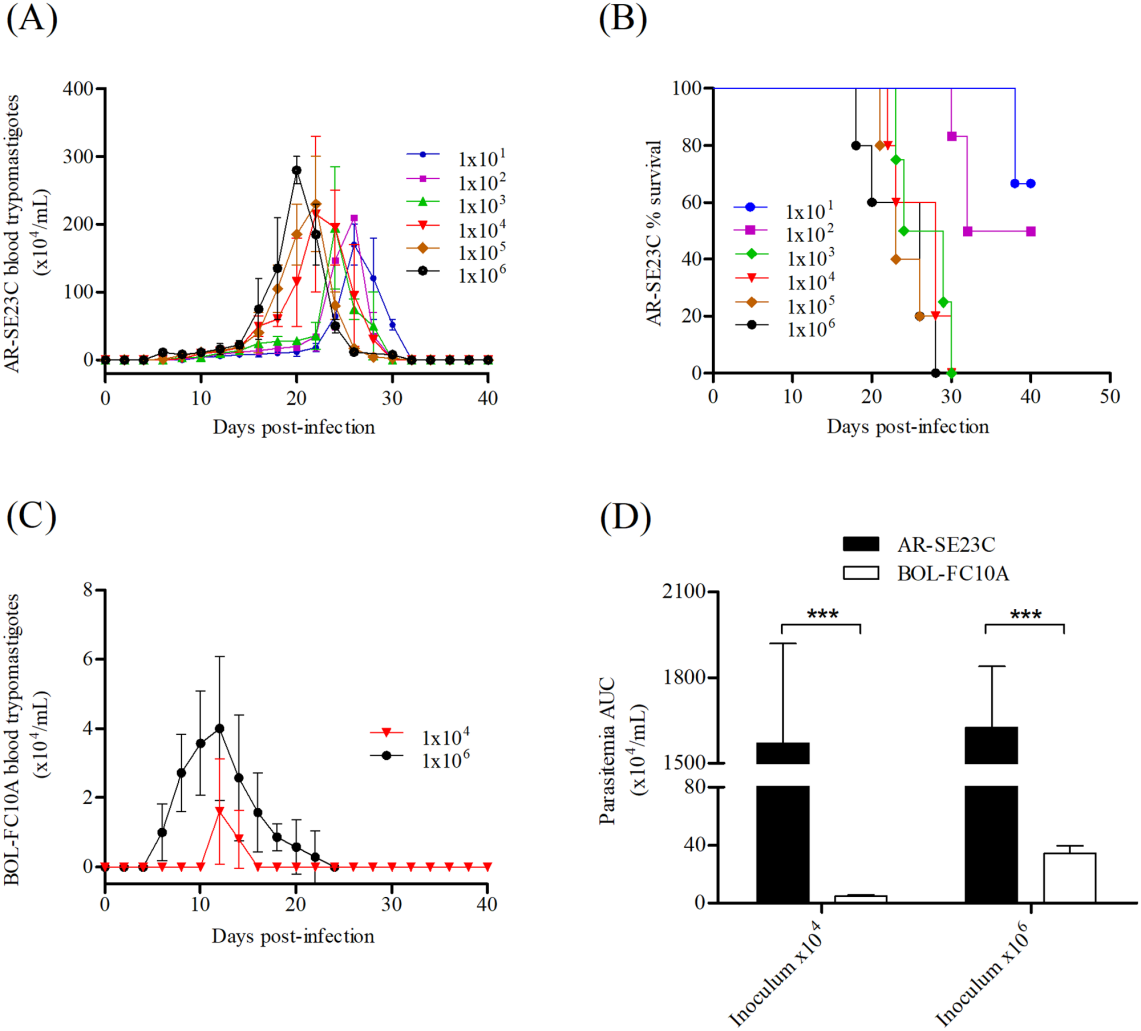
Course of infection in mice inoculated with *Trypanosoma cruzi* AR-SE23C and BOL-FC10A trypomastigotes. **(A)** Parasitemia levels in C3H/He mice inoculated ip with 10^1^ to 10^6^ Vero cell culture-derived AR-SE23C trypomastigotes. **(B)** Mortality was recorded daily and analyzed using log-rank (Mantel Cox) test; P = 0.0056. **(C)** Parasitemia levels in mice infected with 10^4^ and 10^6^ Vero cell culture-derived BOL-FC10A trypomastigotes. **(D)** Graphical representation of parasitemia AUC in mice infected with 10^4^ or 10^6^ AR-SE23C or 10^6^ BOL-FC10A trypomastigotes. ***p<0.001; *t test*.

In order to determine the virulence of TcV BZ-susceptible AR-SE23C and BZ-resistant BOL-FC10A isolates *in vivo*, groups of C3H/He mice were infected with 10^1^ to 10^6^ Vero cell culture-derived trypomastigotes. All mice infected with AR-SE23C had variable levels of parasitemia (Fig 5A). The size of the inoculum correlated with the number of circulating parasites at the peak of parasitemia (r = 0.9429, P = 0.0167; Spearman correlation test), and higher inoculums resulted in earlier parasitemia peaks (r = -0.8827, P = 0.0333; Spearman correlation test). The estimated lethal dose 50 (LD50) of AR-SE23C was 10^2^ parasites (Fig 5B). Circulating parasites were also detected in mice infected with 10^4^ or 10^6^ BOL-FC10A trypomastigotes (Fig 5C), but the area under curve (AUC) and the number of parasites at the peak of parasitemia were significantly lower than that of mice infected with the corresponding AR-SE23C inocula (P = 0.0357 and P = 0.0218, respectively; Fig 5D). No deaths were recorded in the groups of mice infected with BOL-FC10A trypomastigotes. These data indicates that isolate BOL-FC10A is significantly less virulent for C3H/He mice than isolate AR-SE23C, reaffirming the notion of biological intra-type V diversity of *T*. *cruzi*.

**Fig 6:**
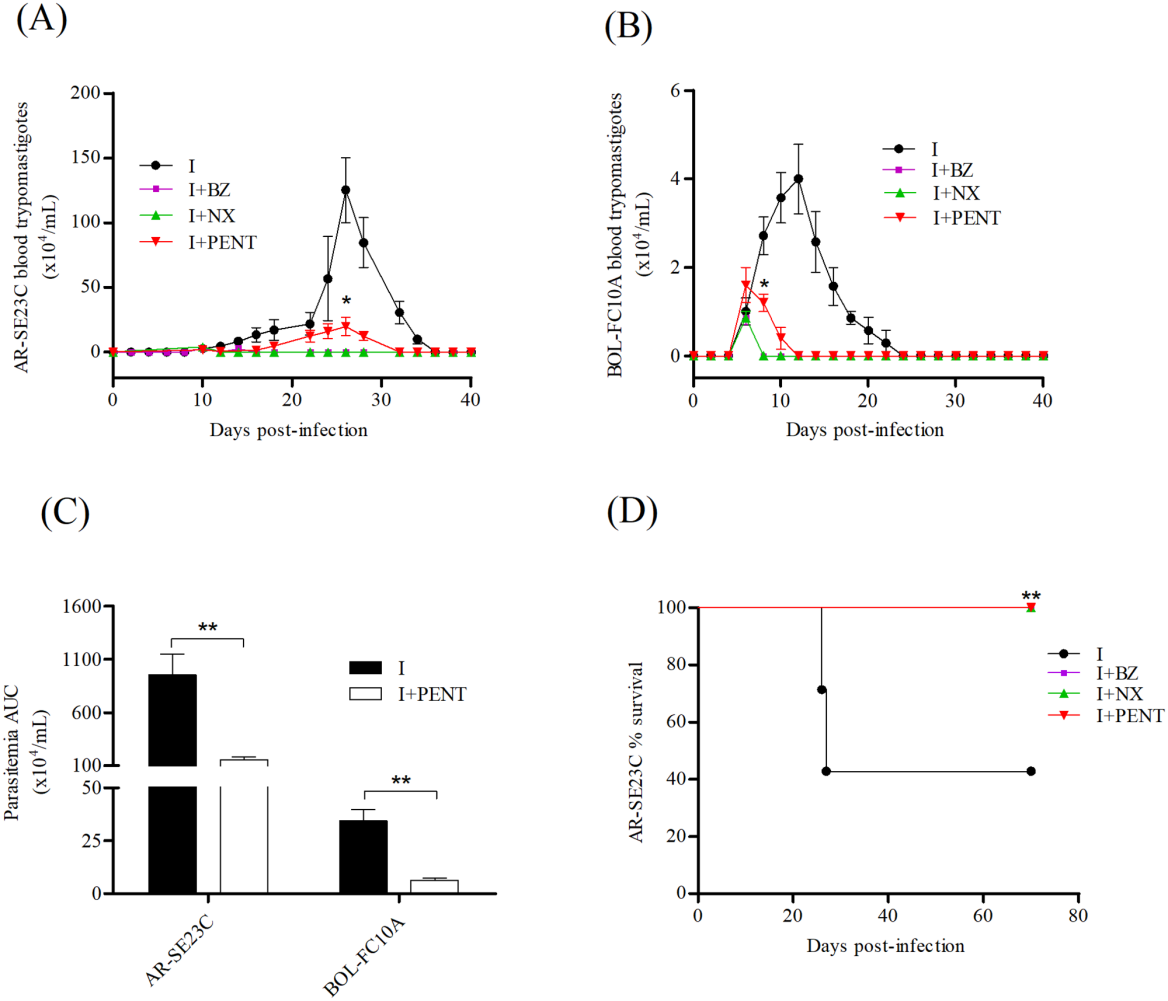
Effect of trypanocidal drugs on the infection of mice with *Trypanosoma cruzi*. Mice were inoculated with 10^2^ AR-SE23C or 10^6^ BOL-FC10A trypomastigotes and treated with BZ (40 doses of 100 mg/kg/d), NX (40 doses of 100 mg/kg/d) or PENT (20 doses of 4 mg/kg/d). Parasitemia curves in mice infected with AR-SE23C **(A)** and BOL-FC10A **(B)**. **(C)** Parasitemia AUC in infected mice treated with PENT. **p<0.01 vs. non-treated infected controls; *t-test*. **(D)** Effect of treatment on survival rate of mice infected with AR-SE23C; **p<0.01 vs. non-treated infected controls; *Mantel-Cox test*.

To investigate the effect of drug treatment *in vivo*, mice were infected with 10^2^ AR-SE23C or 10^6^ BOL-FC10A trypomastigotes and treated with BZ, NX or 4 PENT, starting on the day circulating parasites were first detected in the blood. BZ and NX, but not PENT prevented the increase of patent parasitemia irrespective the infecting parasite (Fig 6A and 6B); nevertheless, PENT was effective to reduce AUC (Fig 6C) and the number of blood parasites at the peak of parasitemia compared to that of non-treated infected controls (p<0.05). The data on the proportion of mice with positive *T*. *cruzi* qPCR and DNA parasite load in blood samples of mice at 70 dpi are shown in Table 4. Notably, the levels of parasite equivalents in non-treated BOL-FC10A-infected controls were significantly higher than that of non-treated AR-SE23C-infected controls with positive qPCR (p<0.05). Treatment of AR-SE23C-infected mice with PENT, but not BZ and NX significantly reduced the proportion of animals with positive qPCR; yet, the three drugs prevented mortality of mice infected with this isolate (Fig 6D). Conversely, BZ and NX but not PENT were effective to reduce the proportion of mice with positive qPCR in BOL-FC10A-infected groups.

**Table 4:** *Trypanosoma cruzi* DNA in blood of mice infected with AR-SE23C or BOL FC10A parasites and treated with benznidazole, nifurtimox or pentamidine.

Treatment	Mice positive/ total (range Eq parasites/mL)	
	AR-SE23C	BOL-FC10A
Non-treated infected control	3/4 (0,15–20,78)	5/5 (90.80–429.0)^§^
Benznidazole	2/5 (0,29–46,34)	0/5*
Nifurtimox	5/5 (0.77–9.11)	0/5*
Pentamidine	1/5* (22.20)	3/5 (262.20–527.0)

Quantitative *T*. *cruzi* DNA amplification was performed at 70 dpi in all groups of mice. Data represent the number of mice with positive *T*. *cruzi* qPCR / total number of mice tested and (range of parasite DNA load in mice with positive qPCR).

*p<0.05 vs. non-treated infected controls; Fisher’s exac*t test*.

^§^p<0.05 vs. non-treated AR-SE23C infected controls; Mann-Whitney test.

## Discussion

In nature, *T*. *cruzi* strains consist of a mixture of parasite subpopulations subjected to selective pressure by different vectors and hosts [20]. The uncovering of biochemical and molecular markers that identify distinct subpopulations within *T*. *cruzi* has led to a great advance in the understanding of parasite population dynamics and epidemiology of the infection [26–30]. TcV is the most frequent genotype reported in infected humans of Argentina, followed by TcVI and TcI [11,31]. In the present study we have established 7 new human-derived *T*. *cruzi* isolates, the majority of which belong to TcV genotype with a minor representation of TcI, suggestive of them being somehow representative of the overall population currently circulating in humans of Argentina. By studying the behaviour of the parasites and performing comprehensive drug sensitivity testing, we were able to expose substantial intra-DTU phenotypic diversity within TcV, similar to that described by Mejia *et al* (and confirmed here) for TcI isolates [32]. In order to maintain the population as close as possible to the natural condition, all assays were performed with parasites obtained from the first 5 passages *in vitro* [33,34]. To favour direct contact between parasites and the drug, we established the parasite drug resistance profile in axenic culture assays. With the purpose of reducing the risk of natural parasite selection by trypanocidal drugs, we enrolled chronically and acutely infected donors with no prior anti-*T*.*cruzi* treatment. Nevertheless, a few of our isolates displayed a resistant phenotype to BZ and NX in drug sensitivity assays. It is possible that these parasites have been exposed to these drugs during circulation in domestic cycle, prior infection of the patients from whom them were retrieved.

The partial effect exerted by the conventional drugs prompted us to extend the study to investigational compounds with anti-*T*.*cruzi* action PENT and DHA, selected on the basis of their different modes of action. The mechanism of action of BZ and NX involves the reduction of the nitro groups to amino groups of the compounds by the action of *T*. *cruzi* nitroreductases, with the formation of toxic radical intermediates and electrophilic metabolites [35]. The activity of BZ against *T*. *cruzi* was proposed to be also mediated by reduced metabolites binding covalently to macromolecules such as lipids, DNA and proteins [36]. Instead, the reduction of NX leading to formation of an unsaturated open-chain nitrile was proposed as an important mechanism for its trypanocidal action [37]. Pentamidine is a synthetic aromatic diamidine widely employed for the treatment of African trypanosomiasis, leishmaniasis and infection by *Pneumocystis carinii* [38,39]. Trypanocidal mechanisms of diamidines include DNA binding, alteration of polyamine transport, inhibition of peptidase activity and interference with normal topoisomerase II. *T*. *cruzi* lacks the enzymes l-arginine decarboxylase and ornithine decarboxylase, required to synthesize putrescine *de novo*, and incorporates the polyamine from the medium through the polyamine transporter TcPAT12, which is blocked by pentamidine [40]. Artemisinin, a sesquiterpene trioxan lactone derived from *Artemisia annua*, and its derivatives are widely used against *P*. *falciparum* and chloroquine-resistant *P*. *vivax*. Anti-malarial action of artemisinins involves the generation of free radicals by cleavage of endoperoxide bonds through the interaction with heme iron, which alter several biochemical pathways within the parasites including the sarco-endoplasmic reticulum PfATPase6 calcium pump (SERCA) [41]. Several membrane-associated calcium ATPases have been described in *T*. *cruzi* and suggested to be responsible, at least in part, for the inhibitory effect of artemisinins on these parasites [42]. In agreement with the results reported by other authors [42], a potent action of PENT against *T*. *cruzi* epimastigotes was observed in our axenic culture assays. However, epimastigote sensitivity assays to DHA revealed that none of the human-derived isolates was susceptible to the compound. Nevertheless, AR-SE23C trypomastigotes resulted susceptible to DHA.

The comparison of the BZ susceptibility/resistance profile between amastigotes and trypomastigotes of the selected isolates reproduced that of epimastigotes (that is, AR-SE23C parasites being more susceptible than BOL-FC10A parasites). Noteworthy, BOL-FC10A epimastigotes and amastigotes were resistant to BZ but susceptible to NX. The higher activity of NX against intracellular amastigotes compared to BZ was previously noted in cell cultures infected with other strains of *T*. *cruzi* [21,43], and suggested to be related to the different modes of action of the two drugs. The pattern of drug sensitivity in our mouse model of infection did not completely translate to that of the parasites *in vitro*, adding further complexity to the development of effective treatments against *T*. *cruzi*. Studies on the susceptibility of *T*. *cruzi* to drugs *in vivo* and *in vitro* carried out by different laboratories have yielded inconsistent results. An example of this is Colombiana strain, which was reported resistant after 20 doses of 100 mg/kg/d BZ *in vivo* by Filardi and Brener [44], but susceptible as intracellular amastigotes *in vitro* [45]. On the other hand, Neal and van Bueren describe that *in vitro* drug sensitivity assays on epimastigotes and intracellular amastigotes did not distinguish between strains of *T*. *cruzi* which were responsive and non-responsive in mice, attributing this phenomenon to the host immune response against *T*. *cruzi* [43]. Conversely, other authors have shown that most drugs that are active against *T*. *cruzi* in the mouse model are also active against epimastigotes [46] or intracellular amastigotes *in vitro* [47]. Besides, *in vitro* sensitivity assays to BZ performed on epimastigotes isolated prior to treatment of patients did not correlate with the clinical therapeutic outcome [48]. The remarkable stage-dependent discrepancies in the sensitivity to drugs found in our study highlight the importance of analysing different developmental stages of the parasite in pharmacological studies of clinically relevant isolates. In this regard, Zingales proposed that natural resistance or sensitivity to drugs can be studied in the three developmental stages of *T*. *cruzi* (epimastigote, trypomastigote and amastigotes) [30].

Finally, we investigated also the possible association of parasite susceptibility/resistance to BZ with the expression of enzymes involved in the anti-redox machinery. *T*. *cruzi* synthesizes trypanothione and several enzymes that neutralize reactive oxygen and nitrogen species as a mechanism of defence against oxidative stress generated during the infection and the metabolism of trypanocidal drugs. As reported for other BZ-resistant *T*. *cruzi* populations, BOL-FC10A epimastigotes underexpressed the Old Yellow enzyme that catalyzes prostaglandin PGF2A synthesis [23,49] and SODB [24], and overexpressed SODA [25]. We also investigated the expression of tryparredoxin 1, known to be involved in oxidative metabolism and protein synthesis and degradation [50], and found it was markedly increased in BZ-resistant BOL-FC10A parasites. Although only 2 isolates were analyzed for anti-redox protein expression, our results support their association with the susceptibility/resistance phenotype of the parasites to BZ.

In summary, herein we demonstrate that *T*. *cruzi* phenotypic diversity levels are particularly high in infected humans of Argentina. Relatively few isolates were analyzed in the study, but the large variety of intra-type responses to drugs suggests that the diversity may be even more extensive than observed here. Such diversity may represent an important difficulty for the development of chemotherapeutic alternatives against Chagas disease. Assuming the present study is applicable to other DTUs, the analysis of such strains will be useful in identifying whether further parasite diversity exist.

## Materials and methods

### Ethics statement

A signed informed consent was obtained from all adult participants and mothers on behalf of their children. All the research was conducted in accordance with the Declaration of Helsinki and the Council for International Organizations of Medical Sciences (CIOMS). The study protocol was approved by the Review Board of Instituto Nacional de Parasitología “Dr. Mario Fatala Chaben”, Administración Nacional de Laboratorios e Institutos de Salud Dr. Carlos G. Malbrán (IRB00006651).

All animals received humane care and study protocols complied with the ARRIVE Guidelines for Reporting Animal Research [51]. The procedures were approved by the Institutional Committee for Care and Use of Laboratory Animals (CICUAL), University of Buenos Aires, School of Medicine, Secretary of Science and Technology, Buenos Aires, Argentina, Resolution No. 704/2013.

### Patients

Chagas disease patients attending the Clinical Facilities of Instituto Nacional de Parasitología “Dr. Mario Fatala Chaben”, Buenos Aires, Argentina and individuals living in endemic areas of Province Santiago del Estero, Argentina, who tested positive for *T*. *cruzi* by conventional serology and had not taken anti- *T*. *cruzi* drugs were invited to participate in the study. Infected individuals with cancer, HIV infection, syphilis, diabetes or autoimmune disorders were excluded from this study. A total of 36 subjects (mean age ± SD = 46.25 ± 12.49 years; 16 males and 20 females) were recruited during the period November 2009—November 2015. The medical history and personal information such as the country of birth and current residency were recorded. The clinical status of participants was assessed by physical examination and clinical testing including electrocardiogram, echocardiography and chest radiography; 25 patients were asymptomatic, 9 patients had ECG abnormalities associated to Chagas heart disease and 2 patients had chronic Chagas dilated cardiomyopathy.

### Parasite isolation

Approximately 15 mL blood were drawn from *T*.*cruzi*- infected patients by venipuncture into dry tubes (Vacutainer; BD Biosciences, Franklin Lakes, NJ, USA) and distributed in glass tubes containing biphasic media (blood-agar with liver infusion tryptose (LIT) as overlay). Samples were cultured at 28°C during 3 months and evaluated monthly for the presence of parasites at a phase microscope (Carl Zeiss, Axiostar plus, Gottingen, Germany). Supernatants from positive hemocultures were transferred to 15 mL culture tubes containing LIT media supplemented with 20 μg/mL haemin, 10% heat-inactivated foetal bovine serum (FBS), 100 U/mL penicillin, and 100 μg/mL streptomycin (complete LIT). Two additional *T*. *cruzi* isolates obtained by hemoculture of children with congenital acute infection during the same period were incorporated in this study [52]. Parasites obtained from the first 5 passages *in vitro* were used in all assays.

### DTU identification

DNA was extracted from 10^8^ axenic culture-derived epimastigotes with *Accuprep Genomic DNA* extraction kit (Bioneer Corporation, Daejeon, Korea) and used for amplification of the non- transcribed spacer of the miniexon (mini-exon genes) and the D7 divergent domain of the 24Sα rRNA gene [18]. Amplification of mini-exon with TC, TC1 and TC2 primers generate products of 300 bp (TcII, TcV and TcVI) and 350 bp (TcI); amplification of the 24Sα rRNA with D71 and D72 primers generates products of 110 (TcI, TcIII, TcV), 125 (TcII and TcVI) and 120–130 bp (TcIV). Reference strains Sylvio X10/4, Y, M5631, CanIII, Mncl2 and CL Brener were used as TcI-TcV controls.

### Epimastigotes drug sensitivity testing

Epimastigote cultures were initiated at a concentration of 10^6^ epimastigotes/mL in complete LIT and exposed to 0.1–80 μg/mL dilutions of BZ, NX, pentamidine isethionate (PENT) or dihydroartemisinin (DHA) in 96-well-plates and incubated at 28°C during 72 h. All compounds were obtained from Sigma-Aldrich (Saint Louis, MO, USA). A stock solution was prepared for each compound in dimethylsulphoxide and diluted in complete LIT media immediately prior to use in the assays; the final concentration of dimethylsulphoxide did not exceed 0.2% (v/v). At the end of each experiment, motile parasites were quantified in a Neubauer’s chamber at a phase microscope. *T*. *cruzi* Sylvio X10/4 epimastigotes were included as susceptibility controls [21]. IC_50_ values were estimated by means of nonlinear dose-response curve analysis using GraphPad Prism 5.0 (GraphPad Software, Inc., La Jolla, CA, USA).

### Transmission electron microscopy

Epimastigotes were washed with PBS, fixed in 2.5% glutaraldehyde and 4% paraformaldehyde in 0.1 M sodium phosphate buffer pH 7.2 and embedded in 2% agarose. Samples were post-fixed in 1% osmium tetroxide—1% potassium ferrocyanide in 100 mM sodium cacodylate buffer at room temperature during 30 minutes, dehydrated in acetone and embedded in Epoxi resin. Ultrathin sections were obtained with an ultramicrotome (Reichert-Jung Ultracut E, Wien, Austria), stained with 2% uranyl acetate and lead citrate, and examined at a transmission electron microscope (Carl Zeiss 109T, Oberkochen, Germany).

### Western blot

Epimastigotes were lysed using 7 M urea, 2 M thiourea, 4% CHAPS detergent, 40 mM Tris, 60 mM 1,4-Dithiothreitol (DTT) (Sigma-Aldrich, Saint Louis, MO, USA), containing the Complete Mini Protease Inhibitor Cocktail (Roche Applied Science, Indianapolis, USA) and centrifuged at 13.000×g for 30 min prior to determination of protein content by the Bradford method. Proteins were separated on 12% SDS polyacrylamide gels (20 μg/well) and transferred onto nitrocellulose membranes. To assure equal protein load in each lane, membranes were stained with Ponceau S. Blots were blocked with skimmed milk powder (5% w/v) in Tris-buffer saline containing 0.02% Tween 20 (TBS-Tween 20) for 3 h. The nitrocellulose membrane was incubated with rabbit polyclonal antibodies anti- mitochondrial superoxide dismutase (SODA; 1:2000), anti- cytosolic superoxide dismutase (SODB; 1:3000), anti- tryparedoxin- 1 (TXN 1; 1:1000) and anti- Old Yellow Enzyme (TcOYE; 1:30000), washed with TBS-Tween 20 and incubated with HRP-conjugated goat-antirabbit IgG diluted 1:2000. Signal was developed using the Chemiluminescence Detection Kit (Thermo Fisher, Waltham, MA, USA). Polyclonal antibodies against *T*. *cruzi* proteins SODA, SODB, and TXN 1 were produced as described [50,53] and TcOYE was a kind gift of Dr. Carlos Robello (Institut Pasteur, Montevideo, Uruguay).

### Amastigote drug sensitivity testing

In preparation for this study, we first tested the infectivity of Vero cell-derived trypomastigotes of selected *T*. *cruzi* isolates for bovine embryo skin and muscle cells (BESM); the poor infectivity of one of the isolates for these cells prevented us from using them for the intracellular multiplication assays (S3 Fig). Then, we tested the infectivity of the parasites for freshly isolated mouse peritoneal macrophages (PMØ). Macrophages were collected from the peritoneal cavity of C3H/HeN mice by lavage with 5 mL of ice-cold RPMI medium, and suspended in 10% FBS-RPMI. Macrophages were seeded onto coverslips (5x10^5^ cells/cover) in 24 well-plates and cultured at 37°C in a 5% CO_2_ incubator during 24 h. Non-adherent cells were removed by washing with RPMI and the cultures infected with Vero cell-derived AR-SE23C or BOL-FC10A trypomastigotes at a ratio of 1:1 during 16 h. After removal of non-internalized parasites, infected cultures were exposed to serial dilutions (0.01–100 μg/mL) of BZ, NX, PENT and DHA in triplicate wells during 72 h prior fixation with methanol and staining with Giemsa. The number of infected cells and intracellular amastigotes/cell was quantified in microphotographs of randomly selected 200x microscopic fields of Giemsa-stained smears (Leica CTR Mic, Germany), using Image Tool software (http://compdent.uthscsa.edu/dig/itdesc.html). A total of 600 cells (200 cells per cover) were analyzed for each experimental condition in 2 independent experiments. IC_50_ values were estimated by means of nonlinear dose-response curves analysis using GraphPad Prism 5.0 Software.

The effect of BZ, NX, PENT and DHA on the viability of peritoneal macrophages was measured in uninfected cell cultures using the resazurin method (Sigma-Aldrich, St. Louis, MO). Briefly, macrophages were plated in 96 well-plates (5×10^5^ cells/well) and incubated with 0.001–5000 μg/mL dilutions of BZ, NX, PENT or DHA at a final volume of 100 μL/well during 72 h at 37 °C in a 5% CO2 incubator. Then, 10 μL of resazurin solution (0.01% in PBS) was added to each well and returned to the incubator for additional 4 h prior to recording of absorbance at 578 and 630 nm. Selectivity index of the compounds (SI) was expressed as the ratio (CC_50_ for macrophages/IC_50_ for *T*. *cruzi* parasites).

### Trypomastigotes drug sensitivity testing

For drug sensitivity assays, Vero cell-derived AR-SE23C and BOL-FC10A trypomastigotes (1x10^6^/mL) were incubated in 10% SFB-RPMI in the presence of serial dilutions (0.01–100 μg/mL) of BZ, NX, PENT and DHA for 24 h at 4°C; untreated parasites were used as controls. The number of motile parasites was determined in a Neubauer chamber at a phase microscope. IC_50_ were calculated as describe above.

### Mice infection

The aim of this experiment was to establish the virulence of selected isolates in the mouse model, with the outcomes measures being the course of parasitemia and mortality. A total of 95 four-week-old female C3H/He mice, obtained from the Animal Breeding Facility of the Instituto Nacional de Parasitología “Dr. Mario Fatala Chabén”. Mice were selected randomly, housed in groups of five per cage at a room temperature of 22°C and provided food and water *ad libitum*. The cages were cleaned and sanitized daily. Mice were inoculated with serial numbers of AR-SE23C and BOL-FC10A Vero cell culture-derived trypomastigotes (10^1^–10^6^) via ip. The physical status, behavior and mortality were monitored by the researcher and recorded daily. The number of circulating parasites was determined in 5 μl of fresh blood samples obtained from the tail in a Neubauer chamber three times a week. Parasitemia AUC was estimated using GraphPad Prism 5.0. To minimize suffering and distress of experimental animals, humane endpoints consisting of weakness and paralysis of the posterior limbs and ataxia that could interfere with eating or drinking [54] were established in the infection protocol at the beginning of the experiment. These signs were used to decide whether to euthanize the animals during the study as a surrogate endpoint for mortality. For termination of animal studies, mice surviving the acute phase of infection were anesthetized by ip administration of a mixture of Ketamine (85mg/kg) and Xylazine (30mg/kg) and euthanized by cervical dislocation at 70 dpi (after the acute phase of infection usually subsides).

To evaluate drug susceptibility *in vivo*, mice were infected with AR-SE23C (10^2^ parasites/mouse) or BOL-FC10A (10^6^/ parasites/mouse) trypomastigotes as described above and treated with 100mg/kg/d BZ (Abarax, ELEA, Argentina) or NX (Lampit, BAYER, El Salvador) during 40 consecutive days by oral gavage, or 4mg/kg/d PENT (Pentamidina, RICHET, Argentina) during 20 consecutive days via intraperitoneal (5 mice/group). Sham-inoculated treated and non-treated infected mice were used as controls. Parasitemia and mortality were determined as mentioned above. Additional blood samples (150 μL/ mouse) were obtained by retro-orbital venipuncture prior euthanasia at 70 dpi for the detection of *T*. *cruzi* DNA by means of qPCR.

### Real time PCR

Mouse blood samples were mixed with an equal volume of guanidine-HCl 6 M, EDTA 0.1 M, pH 8 (GEB), kept at room temperature for 3 days and then stored at 4°C until use. DNA was isolated from 200 μL of GEB using the High Pure PCR Template preparation Kit (Roche, Mannheim, Germany), according to the manufacturers’ protocol. Parasite DNA was amplified using a *T*. *cruzi* satellite DNA sequence of 140 bp flanked by the Sat Fw and Sat Rv oligonucleotides [55] and SYBR GreenER qPCR SuperMix Universal Kit with integrated uracil DNA glycosylase (UDG; Invitrogen, Life Technologies, Grand Island, NY, USA). DNA amplification was performed in an ABI 7500 thermocycler (Applied Biosystems, Carlsbad, CA, USA) in duplicates using 5 μL of extracted DNA as template (~100 ng) in a final volume of 20 μL. DNA extracted from non-infected mice and samples without DNA template were included as controls. qPCR conditions and standard parasite curve for data analysis were performed as previously described [52,56].

### Statistical analysis

The results of assays were expressed as the mean ± SD derived from triplicate observation samples in at least two independent experiments and analyzed using GraphPad Prism. The Kolmogorov-Smirnov normality distribution test was applied to all data. Comparisons between groups were made using unpaired *Student’s t test* (equal variance) or Mann-Whitney test (unequal variance). Comparisons among multiple groups were made using ANOVA followed by Bonferroni or Kruskal-Wallis followed by Dunns. Values of p<0.05 were considered statistically significant.

## Supporting information

S1 Fig Dose-response curves of *Trypanosoma cruzi* epimastigotes. Exponential phase epimastigotes grown in axenic cultures were treated with different dilutions of conventional and investigational trypanocidal compounds during 72 h. At the end of each experiment, motile parasites were quantified using a Neubauer’s chamber. Growth inhibition data represents the mean ± SD of combined results from three independent experiments, each including triplicate samples per dose. IC_50_ doses are listed in Results (Table 2).
(TIF)

S2 Fig Ponceau-S staining. Membranes were stained with Ponceau S to confirm equal loading of epimastigotes samples shown in Fig 4. The molecular size marker (MW) is shown on the nitrocellulose membrane.
(TIF)

S3 Fig *Trypanosoma cruzi* infectivity in mammalian cell cultures. Cultures of peritoneal macrophages and BESM cells were exposed to Vero cell-derived AR-SE23C and BOL-FC10A trypomastigotes during 16 h and cultured for additional 72 h. The number of infected cells was determined using Image Tool Software.
(TIF)
