# Supplementary figures and images for: Phenotypic diversity and drug susceptibility of Trypanosoma cruzi TcV clinical isolates
Source: PLoS One. 2018 Sep 5;13(9):e0203462. doi: 10.1371/journal.pone.0203462 (PMC6124804; doi:10.1371/journal.pone.0203462)

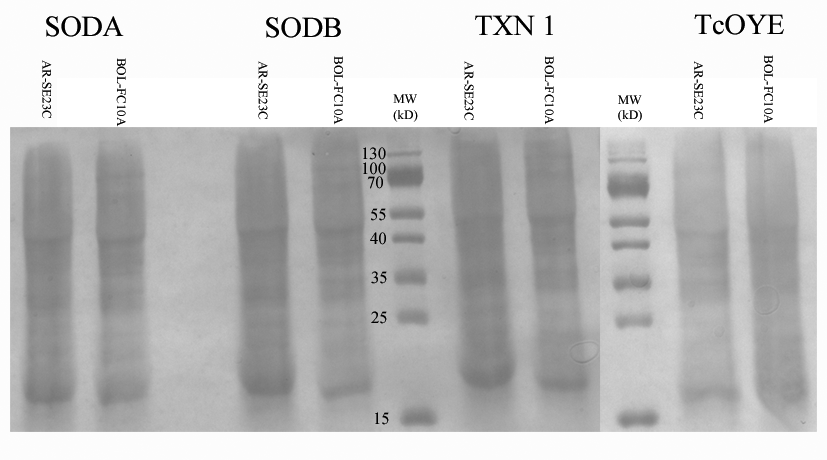

Supplement: S2 Fig — Membranes were stained with Ponceau S to confirm equal loading of epimastigotes samples shown in Fig 4. The molecular size marker (MW) is shown on the nitrocellulose membrane. (TIF) [file pone.0203462.s002.tif]
